# Clinical, pathological and genetic features as well as follow-up of 68 patients with late-onset Pompe disease: a single-center retrospective study

**DOI:** 10.3389/fnut.2026.1797345

**Published:** 2026-04-28

**Authors:** Duoling Li, Yixin Shi, Hanhan Sun, Na Zhang, Hang Su, Wei Li, Dandan Zhao, Bing Wen, Yuying Zhao

**Affiliations:** 1 Department of Neurology, Shandong Key Laboratory of Mitochondrial Medicine and Rare Diseases, Research Institute of Neuromuscular and Neurodegenerative Diseases, Qilu Hospital of Shandong University, Jinan, Shandong, China; 2 Shandong Key Laboratory of Mitochondrial Medicine and Rare Diseases, Research Institute of Neuromuscular and Neurodegenerative Diseases, Cheeloo College of Medicine, Shandong University, Jinan, Shandong, China; 3 Department of Neurology, Shanxian Central Hospital, Heze, Shandong, China; 4 Department of Neurology, Shandong Provincial Hospital Affiliated to Shandong First Medical University, Jinan, Shandong, China

**Keywords:** enzyme replacement therapy (ERT), follow up, GAA gene, late-onset Pompe disease (LOPD), muscle pathology

## Abstract

**Introduction:**

Pompe disease is a muscular lysosomal storage disorder characterized by autosomal recessive inheritance and caused by deficiency of the acid alpha-glucosidase (GAA) enzyme. Late-onset Pompe disease (LOPD) exhibits heterogeneous clinical presentations, which are influenced by the type of GAA mutation and residual enzyme activity.

**Methods:**

In this study, we conducted a retrospective analysis of 68 Chinese LOPD patients over a 17-year period at a single center to delineate the real-world disease status and survival outcomes.

**Results:**

Among the 47 patients who received enzyme replacement therapy (ERT), the mortality rate was 6.4%, compared to 57.1% in the 21 patients who did not receive ERT. Muscle pathology analysis revealed that glycogenin accumulation appears earlier than autophagy marker, but this finding is exploratory and requires validation in larger studies. The severity of muscle pathology correlated with lower body mass index (BMI), shorter 6-min walk test (6MWT) and spinal curvature abnormalities. The most frequent *GAA* gene mutation identified was c.2238G > C (p.W746C), present in 43.3% of patients. In an exploratory subgroup analysis (*n* = 10), patients who initiated ERT shortly after diagnosis exhibited greater improvements in muscle strength and 6MWT results compared to those who started treatment later; however, the small sample size precludes definitive conclusions.

**Conclusion:**

In summary, our exploratory findings hypothesize that glycogenin may be an early pathological marker, but this requires prospective validation. ERT was associated with higher survival probability in this cohort, although survival bias limits causal inference. Earlier ERT initiation showed an association with better functional outcomes in a small subgroup. Lower BMI, shorter 6MWT, and spinal curvature abnormalities correlated with more severe muscle pathology in univariate analyses. All findings should be interpreted as hypothesis-generating due to study limitations.

## 1 Introduction

Pompe disease, also referred to as glycogen storage disease type II (GSD II, OMIM #232300), is an autosomal recessive lysosomal storage disorder characterized by a deficiency of the enzyme acid alpha-glucosidase (GAA) (1). This deficiency leads to the accumulation of glycogen within lysosomes because GAA is responsible for hydrolyzing lysosomal glycogen; its absence impairs degradation, causing progressive storage. The resultant accumulation predominantly affects skeletal and cardiac muscle, giving rise to a spectrum of clinical manifestations that range from severe infantile forms to milder late-onset variants (2–7). The considerable variability in clinical presentation can be largely attributed to the nature and severity of the underlying genetic mutations, as well as the age at which symptoms manifest; both factors significantly influence disease progression and patient outcomes.

Current therapeutic options for Pompe disease primarily include enzyme replacement therapy (ERT), which has demonstrated efficacy in improving clinical outcomes for many patients. ERT aims to replenish the deficient enzyme, thereby facilitating glycogen degradation and alleviating associated symptoms. However, the effectiveness of ERT can vary considerably and is often influenced by the timing of its initiation; earlier treatment is generally correlated with more favorable outcomes (8–11). Despite advancements in ERT, several challenges persist—particularly concerning accessibility, adherence to treatment protocols, and management of related complications (12).

Recent literature underscores significant disparities in mortality rates associated with Pompe disease across various geographical regions. Studies indicate mortality rates ranging from 15 to 30% among European cohorts, with even higher figures reported in certain Asian populations due to delayed diagnosis and limited access to ERT (13). Notably, there is a scarcity of comprehensive mortality data from mainland China, although studies from Taiwan have suggested a mortality rate of approximately 25% among affected individuals (14). This gap underscores the necessity for further research to elucidate the clinical characteristics and survival outcomes of Pompe disease within diverse populations.

Research has elucidated multiple pathological features associated with Pompe disease, including glycogen accumulation, autophagic dysfunction, and inflammation (14). Histological examinations have revealed significant pathological changes in muscle biopsies, characterized by distinct staining patterns that indicate the extent of glycogen storage and lysosomal activity (15). Additionally, there is a recognized need for comprehensive studies linking clinical manifestations with pathological findings, particularly concerning the progression from early to late stages (16). A deeper understanding of the natural history and pathological features of the disease is essential for improving patient outcomes and guiding future research directions in this field.

This study employs a retrospective cohort design, allowing for an in-depth analysis of clinical data from Pompe disease patients treated at a single center. This methodology provides a unique opportunity to compile extensive longitudinal clinical information, facilitating a nuanced examination of mortality rates and disease progression. The primary objective is to descriptively compare survival between LOPD patients who received enzyme replacement therapy (ERT) and those who did not, while acknowledging significant survival and selection bias that preclude causal inference. The secondary objectives are to characterize the clinical, pathological, and genetic features of LOPD in a single-center Chinese cohort. Exploratory analyses include: (1) the association between timing of ERT initiation and changes in 6MWT and muscle strength (small subgroup, *n* = 10); (2) univariate correlations between muscle pathology severity and clinical variables (BMI, 6MWT, spinal curvature). All findings should be interpreted as hypothesis-generating due to the retrospective design and study limitations (16).

By focusing on a single-center cohort, this investigation seeks to contribute valuable insights into the management of Pompe disease, potentially informing clinical practice and guiding future research efforts. Understanding the relationship between early intervention, treatment adherence, and patient outcomes is critical for enhancing care strategies and improving quality of life for individuals affected by this condition. Ultimately, the findings from this study could play a pivotal role in shaping clinical guidelines and optimizing therapeutic approaches for Pompe disease (15).

## 2 Methods

### 2.1 Participants/patients

We performed a retrospective review of medical records for patients diagnosed with LOPD at the Research Institute of Neuromuscular and Neurodegenerative Diseases, Qilu Hospital, Shandong University, between 2004 and 2024. A total of 81 patients with Pompe disease were identified, of which 68 met the criteria for LOPD and were followed up for a period of maximum 17 years.

This study is both real-world and retrospective in nature. The diagnosis of LOPD was established according to the European Pompe Consortium guidelines, requiring at least two out of three criteria: (1) identification of allelic variants in the GAA gene, with at least one variant being previously reported as pathogenic; (2) documentation of glycogen storage via PAS staining on muscle biopsy; (3) reduced enzymatic activity in leukocytes, fibroblasts, or skeletal muscle. (4) With presentation after 12 months of age or presentation at ≤12 months without cardiomyopathy.

Respiratory function was assessed using forced vital capacity (FVC) measured in the sitting position. Data on supine FVC, diaphragmatic weakness (e.g., fluoroscopy or ultrasound) were not systematically collected and thus are not reported.

Muscle strength was evaluated using the Medical Research Council (MRC) grading scale. Follow-up assessments were conducted annually through outpatient visits and telephone consultations. Data collection during clinic visits adhered to a standardized template. Ethical approval was obtained from the Medical Ethics Committee of Qilu Hospital, Shandong University, and all participants provided written informed consent.

Statistical analyses were conducted using SPSS for Windows (Version 27). Survival analysis was performed using the Kaplan–Meier method. Categorical variables were analyzed using Chi-square tests, while numerical variables were assessed using t-tests. Point-biserial correlation analysis was applied to test the significance between dichotomous and continuous variables, while Spearman correlation analysis was employed to evaluate the significance between ordered categorical and continuous variables. Two-sided *p*-values were calculated, with *p* < 0.05 indicating statistical significance. Due to limited sample size and missing data, multivariate regression analyses were not feasible. All reported correlations are unadjusted and should be considered exploratory.

### 2.2 Muscle pathology

Open muscle biopsies of the biceps brachii were performed on all patients under local anesthesia. Muscle samples were rapidly frozen in isopentane pre-cooled with liquid nitrogen and stored at −80 °C. For histological examination, serial frozen sections (8 μm) were stained using hematoxylin–eosin (H&E), Oil Red O (ORO), succinate dehydrogenase (SDH), modified Gomori trichrome (mGT), nicotinamide adenine dinucleotide-tetrazolium reductase (NADH-TR), cytochrome c oxidase (COX), periodic acid-Schiff (PAS), adenosine triphosphatase (ATPase at pH 4.3 and pH 10.4), and acid phosphatase (ACP). Immunohistochemical studies were conducted on serial frozen sections (5 μm) incubated with antibodies against dystrophin, dysferlin, *α*-sarcoglycan, *β*-sarcoglycan, *γ*-sarcoglycan, *δ*-sarcoglycan, caveolin-3, GN, lAMP1, LC3B,P62 and STBD1. Electron microscopic analysis was performed as previously described (17).

For immunofluorescence staining, samples were incubated overnight at 4 °C with two primary antibody solutions simultaneously: Glycogenin-1 (1,400, SC-271109, Santa Cruz Biotechnology, USA) and LAMP1 (lysosome-associated membrane protein 1, 1:200, ab24170, Abcam, UK). Following this, the samples were incubated with fluorescently labeled secondary antibodies for 1 h at 37 °C in the dark. To prevent fluorescence quenching, samples were then treated with DAPI antifade mounting medium at room temperature for 5 min. Imaging was conducted using an Olympus fluorescence confocal microscope. For colocalization analysis, over 50 muscle fibers from multiple fields of view were selected. The number of colocalized muscle fibers was manually counted, and the percentage of colocalization was quantified.

### 2.3 Molecular studies

Prior to 2012, genomic DNA-based PCR amplification and sequence analysis of the GAA gene were performed. Genomic DNA was extracted from frozen muscle biopsy specimens using a genomic DNA extraction kit (Tiangen, PR China). PCR amplifications of all coding regions and intron-exon boundaries were carried out under standard conditions, followed by direct bidirectional sequencing. The initiating ATG codon was designated as base pairs 1–3, and the initiating methionine was numbered as amino acid 1. For more recent cases, next-generation sequencing with target area capture technology and high-throughput sequencing was utilized (RunningGene Inc., Beijing, China).

## 3 Results

### 3.1 Clinical and biochemical features

The age of onset for the 68 LOPD patients ranged from 1 to 45 years, with a mean of 16.0 years and a sex ratio of approximately 0.84:1 (31 females to 37 males). Among these patients, nine exhibited an onset age earlier than 3 years, none of whom experienced cardiac involvement or early mortality; consequently, these nine cases were categorized as early childhood-onset LOPD. At the time of diagnosis, the average disease duration was 3.9 years, with the longest recorded duration being 26 years. Notably, 21 out of 68 patients (30.1%) experienced delayed diagnosis exceeding 5 years. The most prevalent clinical manifestation was proximal muscle weakness, particularly in the lower limbs, observed in 65 out of 68 patients (95.6%). Only two patients presented with asymmetrical muscle weakness. Of the 37 patients who underwent neck muscle examination, 25 (67.6%) demonstrated neck muscle weakness. Additional extramuscular symptoms included diarrhea in 25 cases (36.8%), scoliosis in 23 cases (33.8%), spinal rigidity in nine cases (13.2%), and cerebral vascular abnormalities in six cases (8.8%). The initial symptoms at onset varied, with proximal lower limb weakness reported in 38 patients, respiratory failure in 16, elevated creatine kinase (CK) detected on laboratory testing in seven, and scoliosis or spinal rigidity in five. Notably, no patient presented with proximal upper limb weakness as an initial symptom.24 out of 68 patients (35%) required noninvasive ventilator-assisted ventilation at night or during the day.

GAA enzyme activity was assessed in leukocytes using dried blood spots (DBS) in 57 patients, revealing reduced activity with a mean of 0.77 umol/L/h (reference range: 0.0–3.38 umol/L/h). Among 40 eligible patients, median baseline FVC (forced vital capacity) was 46.9 (range: 15.5–122.7). Laboratory tests indicated serum creatine kinase (CK) levels with a mean of 823.6 U/L (range: 46–2,431 U/L; reference range: 26–178 U/L), which is approximately 4.6 times the upper limit of normal on average. CK levels were within normal limits in six patients (8.8%).

Serum lactate dehydrogenase (LDH) levels were measured in 35 patients, averaging 422.3 U/L (range: 230–1,255 U/L; reference range: 120–230 U/L). Conversely, serum creatinine (Cr) levels were lower than normal in 34 patients, averaging 36.2 μmol/L (range: 23–108 μmol/L; reference range: 62–115 μmol/L). BMI and 6MWT results are summarized in Table 1. Briefly, the mean BMI was 17.9 (range 9.8–25.3), with 55.8% of patients underweight. The mean 6MWT distance was 376.5 meters (range 38–544 meters).

**Table 1:** Summary of clinical and biochamical features of 68 patients with late-onset Pompe disease at diagnosis.

Clinical features		Number
Sex	Male	37/68 (54.4%)
	Female	31/68 (45.6%)
Onset age (years)	Onset age, mean(range)	16.0 (newborn-45)
	1.0–2.9	9/68 (13.2%)
	3.0–10.9	15/68 (22.1%)
	11.0–20.9	20/68 (29.4%)
	21.0–30.9	18/68 (26.5%)
	≥31.0	6/68 (8.9%)
Disease duration (year)	Disease duration, mean (range)	3.9 (0–26.0)
	<1.0	24/68 (35.3%)
	1.0–2.9	13/68 (19.1%)
	3.0–4.9	10/68 (14.7%)
	≥5.0	21/68 (30.1%)
Muscle weakness	Proximal lower limbs	65/68 (95.6%)
	Neck muscle	25/37 (67.6%)
	Unsymmetrical	2/68 (2.9%)
Extra muscular symptoms	Diarrhea	25/68 (36.8%)
	Scoliosis	23/68 (33.8%)
	Spinal rigidity	9/68 (13.2%)
	Cerebral vascular abnormality	6/68 (8.8%)
Onset symptoms	Proximal lower limbs weakness	38/68 (55.9%)
	Respiratory failure	16/68 (23.6%)
	HyperCKemia	7/68 (10.3%)
	Spinal malformation	5/68 (7.4%)
	Unable to recall	2/68 (2.9%)
	Proximal upper limbs weakness	0/68 (0.0%)
GAA enzyme activity (umol/L/h)	*n* = 57	0.77 (0.0–3.38)
FVC (%)	*n* = 40	46.9 (15.5–122.7)
CK (U/L), mean (range)	*n* = 68	823.6 (46–2,431)
LDH (U/L)	*n* = 35	422.3 (230–1,255)
Serum Cr (rum Cr5)	*n* = 34	36.2 (23–108)
BMI	*n* = 51	17.9 (9.8–25.3)
6MWT (m)	*n* = 38	376.5 (38–544)

FVC, forced vital capacity; CK, serum creatine kinase (normal value: 26–178 mmol/L); LDH (normal value: 120–230 U/L); Serum Cr, (62-115μmol/L).

BMI, body mass index; 6MWT, 6-min walk time test.

### 3.2 Survival curve

Survival time was defined as the interval from disease onset to death. Because only 15 of 68 patients (22.1%) died during follow-up, the median survival for the entire cohort was not reached (cumulative survival probability 77.9% at last follow-up). Among the 15 deceased patients, the median time from onset to death was 5.8 years (range: 0.3–13.5 years). In a sensitivity analysis excluding nine patients with onset before age 3 years, the cumulative survival probability at last follow-up was 81.4%, and the median survival remained not reached. Among the deceased patients in this subgroup, the median time from onset to death was 7.2 years (data not shown).

**Figure 1:**
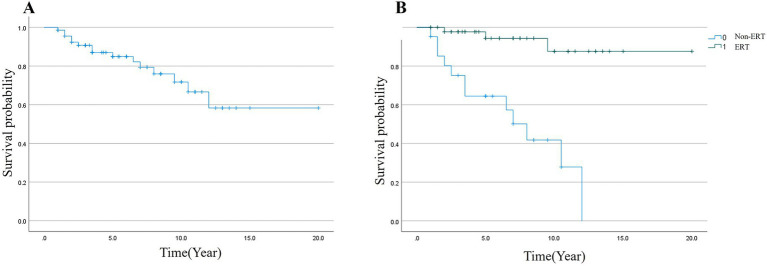
Survival analysis by the Kaplan–Meier method. **(A)** The cumulative survival probability was 72.0% in 75 LOPD cases until September, 2024; **(B)** The cumulative survival probability was significantly lower in non-ERT group (37.0%, blue curve) than that in ERT group (91.7%, green curve, **(B)**, *p* < 0.001).

The overall mortality rate was 22.1%. Among the 47 patients receiving ERT, the mortality rate was 6.4% (3/47), compared to 57.1% (12/21) in the 21 patients not receiving ERT. The cumulative survival probability was 77.9% overall (53/68 patients, Figure 1A). In descriptive comparison, the observed survival probability was higher in the ERT group (93.6%, 44/47 patients) than in the non-ERT group (42.9%, 9/21 patients; *p* < 0.001 for the difference in Kaplan–Meier curves, Figure 1B). However, due to survival and selection bias (ERT became available only after 2017 and reimbursed after 2020), this comparison should not be interpreted causally.

### 3.3 Pathology features

**Figure 2:**
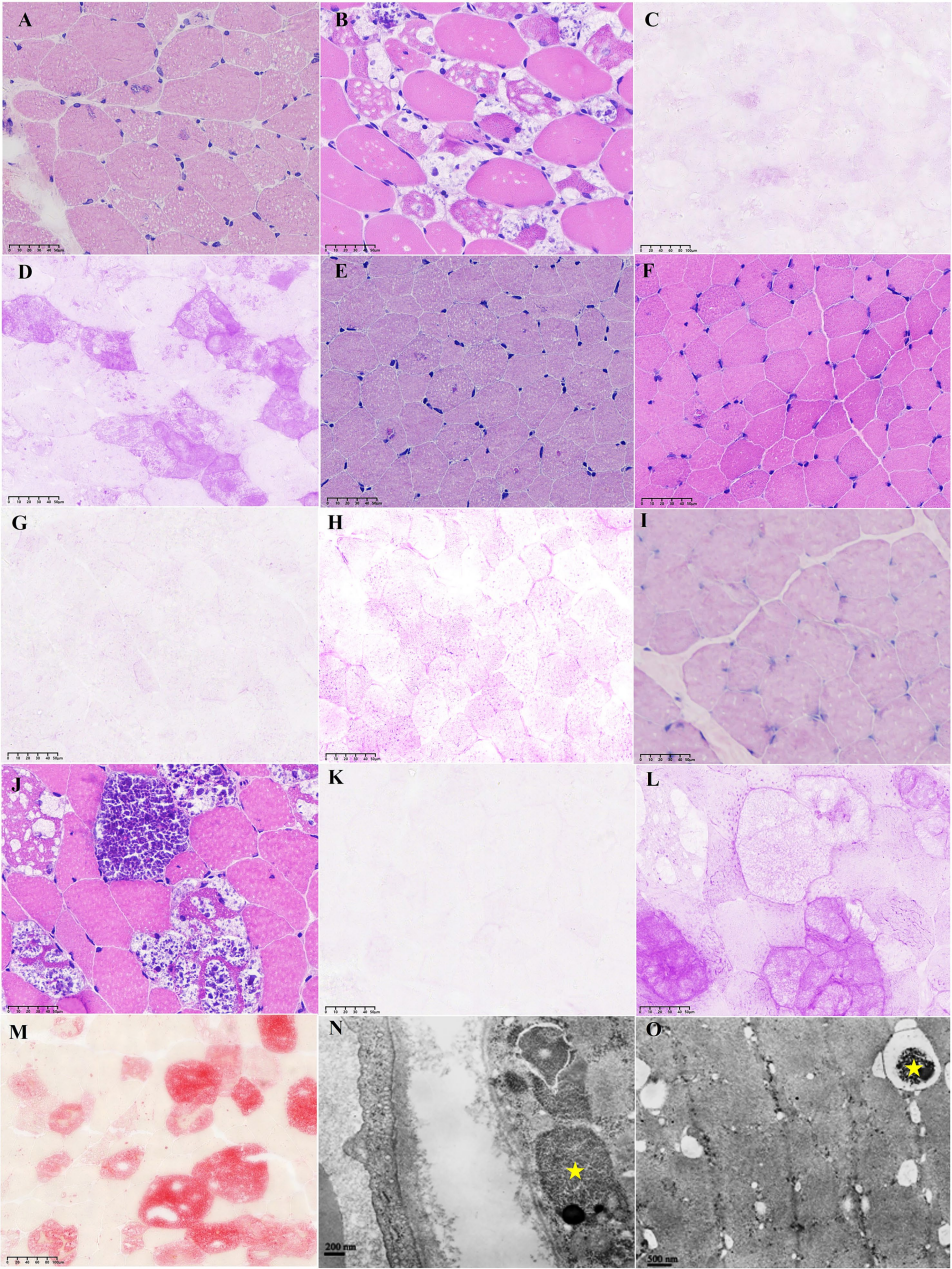
Pathological features of late-onset Pompe disease. **(A)** a variation in fiber size, small vacuoles and scattered basophilic particles in muscle fibers (H&E, first biopsy, patient 4); **(B)** more and larger vacuoles (H&E, second biopsy, patient 4); **(C)** mild glycogen storage (PAS, first biopsy, patient 4); **(D)** severe glycogen storage (PAS, second biopsy, patient 4); **(E)** small vacuoles and scattered basophilic particles (H&E, first biopsy, patient 9); **(F)** similar to G (H&E, second biopsy, patient 9); **(G)** slight glycogen storage (PAS, first biopsy, patient 9); **(H)** more glycogen storage (PAS, second biopsy, patient 9); **(I)** tiny vacuoles(H&E, first biopsy, patient 20); **(J)** large vacuoles and many basophilic particles (H&E, second biopsy, patient 20); **(K)** no glycogen storage (PAS, first biopsy, patient 20); **(L)** obvious glycogen storage (PAS, second biopsy, patient 20); **(M)**strong positive (ACP, second biopsy, patient 20); **(N)** the aggregation of glycogen particles (electron microscopy, star); **(O)** autotropic lysosome that phagocytes glycogen particles (electron microscopy, star).

We conducted a comprehensive analysis of muscle pathology in 35 patients with LOPD (Figure 2). H&E staining revealed significant variability in fiber size, along with numerous vacuoles and small basophilic particles within muscle fibers (Figures 2A,B,E,F,J). MGT staining highlighted fibers containing atypical rimmed vacuoles (RVs) and cytoplasmic bodies. PAS staining demonstrated varying degrees of glycogen accumulation, ranging from mild to severe across different patients (Figures 2C,D,G,H,K,L). ATPase (pH 10.4) staining indicated type II fiber atrophy, with most vacuoles localized in these fibers. ACP staining showed strong positivity (Figure 2M).

In three patients, muscle biopsies were obtained twice prior to the initiation of ERT. For Patient 4, muscle pathology significantly deteriorated over a 7-year period (Figures 2A,C vs. B,D), coinciding with her pregnancy. Specifically, her 6MWT performance declined from 390 meters to 336 meters, iliopsoas muscle strength decreased from grade 3 to 2, and FVC reduced from 64.9 to 58.9%. However, for Patient 9, no significant changes were observed on H&E staining between the two samples taken 5 years apart (Figures 2E vs. F), although his iliopsoas muscle strength decreased from grade 4.5 to 4. For Patient 20, the first biopsy was relatively normal, while the second biopsy, taken 17 years later, exhibited typical glycogen storage (Figures 2I,K vs. J,L). During this period, the patient’s iliopsoas muscle strength also decreased from grade 4 to 3.5. Electron microscopy demonstrated the aggregation of glycogen particles between myofibrils, resulting in the formation of a “glycogen lake” (Figure 2N, star). Additionally, autophagic lysosomes engulfing glycogen particles were observed (Figure 2O, star).

**Figure 3:**
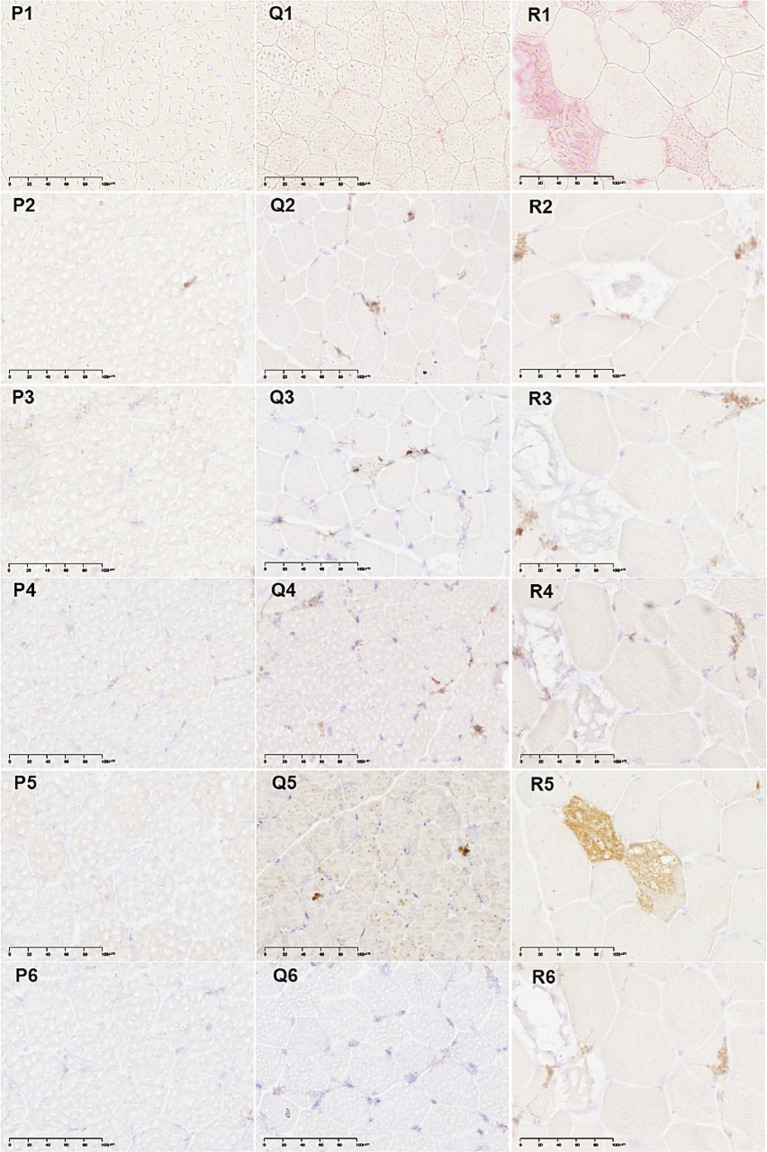
Immunohistochemical analysis of late-onset Pompe disease. First muscle biopsy of patient 9 (P1–6); second muscle biopsy of patient 9 (Q1–6); muscle biopsy of patient 20 (R1–6); ACP staining (P1,Q1,R1); GN (glycogenin) staining (P2,Q2,R2);LAMP1 staining (P3,Q3,R3); LC3B staining (P4,Q4,R4);P62 staining (P5,Q5,R5);STBD1 staining (P6,Q6,R6).

Immunohistochemical analysis revealed that, during the early stage of disease progression in patient 9 (Figures 3P1–5), only a limited number of glycogenin-positive muscle fibers were detected (Figure 3P2). In contrast, autophagy-related markers, including LAMP1, LC3B, and P62, were all negative (Figures 3P3–6), as was ACP staining (Figure 3P1). As the disease progressed, a second biopsy from patient 9 demonstrated positive expression of autophagy-related proteins (LAMP1, LC3B, and P62; Figures 3Q3–6) and ACP (Figure 3Q1), while more glycogenin-positive muscle fibers were observed (Figure 3Q2). These findings raise the hypothesis that glycogenin may be an early pathological marker for Pompe disease, while autophagic accumulation may occur predominantly in the middle to late stages. Given the very limited number of longitudinal samples (*n* = 3), this hypothesis requires validation in larger prospective studies. In patient 20, whose muscle pathology represents an advanced disease stage (Figures 3R1–6), both glycogenin and autophagy markers (LAMP1, LC3B, P62, and ACP) exhibited strong positivity.

The severity of muscle pathology in LOPD ranged from mild to extremely severe (Table 2; criteria for severity are provided in Supplementary Figure S1). Therefore, we performed a correlation analysis between muscle pathology severity (including fiber size variability, presence of basophilic particles on H&E, vacuolated fibers, positive PAS staining, RVs, ACP staining and LAMP1 and Glycogenin-1 colocalization ratio) and clinical features such as disease duration at the time of biopsy, GAA enzyme activity, FVC, BMI, CK levels, 6MWT, iliopsoas muscle strength, and changes in spine curvature. The results indicated that lower BMI correlated with more severe PAS staining and a higher number of vacuoles in muscle fibers (*p* < 0.05). In patients with spinal curvature abnormalities, PAS staining tended to be more severe, and ACP staining was more intense (p < 0.05). The higher the proportion of LAMP1 and Glycogenin-1 colocalization, the shorter the 6MWT of patients (*p* < 0.05).

**Table 2:** The muscle pathology of late-onset Pompe disease.

No.	Fibers size variation	Small basophilic particles on H&E staining	Vacuole fibers	Positive PAS staining	RVs	ACP	P62
01	++	++	++	+++	+	NA	NA
02	+	+	+	+	−	+	NA
03	++	+	++	+++	−	+++	NA
04	++	+	+	+	+	NA	NA
07	+++	+++	++	++	−	+++	NA
08	+++	+	+	+	+	+	NA
09	++	+	+	+	+	++	NA
12	+	++	++	−	−	NA	NA
14^#^	+++	+++	++	+	+	NA	NA
16	++	+++	++	++	+	NA	NA
17a	++	+	+	++	+	++	+
19b	++	++	+++	++	+	NA	NA
20b	−	−	+	−	−	NA	NA
23d	++	+	+	++	+	+++	+++
26e	+++	+++	+++	+++	−	NA	NA
30 g^#^	+	+	+++	−	+	NA	NA
33^#^	++	+	+	+	−	+++	NA
34^#^	+++	+++	+++	+	+	NA	NA
39^#^	+++	+++	+++	++	−	+++	NA
40^#^	+++	+++	+++	++	−	NA	NA
43	++	+++	++	+++	+	+++	+++
45	+	+	+	+	−	NA	+
46	+	+	+	++	+	NA	+
47	+	++	++	++	+	+++	++
48	+++	+	+	+	−	+	+
53	++	++	+++	+++	+	+++	NA
59	++	+++	+++	+++	+	+++	+++
61	+	+	+	+	−	NA	NA
62	+	++	++	+	+	+++	NA
67^#^	++	++	+++	+++	+	+++	+++
70	+	+	+	++	+	+	+
71	+	++	++	+	+	+	−
73	++	+	+	+++	−	+++	++
78	++	++	++	++	−	++	+
80	++	+	++	++	−	++	++

NA, not available; ND, not detected; ^#^, Passed away; +, mild; ++, moderate; +++, severe.

### 3.4 Genetic features

Genetic analysis revealed that 67 patients with LOPD carried variants in the *GAA* gene, of which 63 were compound heterozygotes, one was homozygous, and three harbored only a single variant (Table 3). The severity of these variants was predicted according to the guidelines outlined by Kroos et al. (15), where classification was based on expression studies and variant type (18, 19).

**Table 3:** GAA gene variations in 68 patients with late-onset Pompe disease.

NO.	Variant 1	Predicted severity*	Variant 2	Predicted severity*	Variant 3	Predicted severity*
01	c.546G > A p.T182T	Potentially mild	c.2662G > T p.E888*	Very severe		
02	c.2238G > C p.W746C	Potentially mild	c.2662G > T p.E888*	Very severe		
03	c.1562A > T p.E521V	Unknown	c.1327-7 T > A *p.?*	Unknown		
04	c.546G > T p.T182T	Potentially mild	c.1735G > A p.E579K	Potentially less severe		
05	c.2105G > A p.R702H	Potentially mild	c.2238G > C p.W746C	Potentially mild		
06	c.1465G > A p.D489N	Potentially less severe	c.2238G > C p.W746C	Potentially mild		
07	c.837G > C p.W279C	Potentially less severe	c.2238G > C p.W746C	Potentially mild		
08	c.1844G > T p.G615V	Potentially less severe	c.2173C > T p.R725W	Less severe		
09	c.2237G > A p.W746*	Very severe	c.2238G > C p.W746C	Potentially mild		
10	c.1634C > T p.P545L	Less severe	c.2662G > T p.E888*	Very severe		
11	c.1139C > T p.S380F	Very severe	c.1409A > G p.N470S	Unknown		
12	c.875A > G p.Y292C	Potentially mild	c.1622C > T p.P541L	Very severe		
13	c.1280 T > C p.M427T	Unknown	c.2238G > C p.W746C	Potentially mild		
14^#^	c.1669A > T p.I557F	Very severe	c.2132C > G, p.T711R	Very severe		
15	c.1634C > T p.P545L	Less severe	c.2185delC p.L729fs*35	Very severe		
16	c.2237delG p.W746 fs*17	Very severe	c.1634C > T p.P545L	Less severe		
17a	c.1822C > T p.R608*	Very severe	c.827_845delTCACCCTGTGGAACCGGGA p.I276T*fs* *31	Very severe	c.2240G > A p.G747E	Potentially less severe
18a	c.1822C > T p.R608*	Very severe	c.827_845delTCACCCTGTGGAACCGGGA p.I276T*fs* *31	Very severe	c.2240G > A p.G747E	Potentially less severe
19b	c.2105G > A p.R702H	Potentially mild	c.2238G > C p.W746C	Potentially mild		
20b	c.2105G > A p.R702H	Potentially mild	c.2238G > C p.W746C	Potentially mild		
21c	c.1562A > T p.E521V	Unknown	c.1814G > A p.G605D	Unknown		
22c	c.1562A > T p.E521V	Unknown	c.1814G > A p.G605D	Unknown		
23d	c.241C > T pQ81*	Very severe	c.2238G > C p.W746C	Potentially mild		
24d	c.241C > T pQ81*	Very severe	c.2238G > C p.W746C	Potentially mild		
25d	c.241C > T pQ81*	Very severe	c.2238G > C p.W746C	Potentially mild		
26e	c.692 + 5 G > A *p.?*	Less severe	c.796C > T p.P266S	Potentially mild		
27e^#^	c.692 + 5 G > A *p.?*	Less severe	c.796C > T p.P266S	Potentially mild		
28f	−32-13 T > G *p.?*	Potentially mild	c.1839G > A p.W613*	Very severe		
29f	−32-13 T > G *p.?*	Potentially mild	c.1839G > A p.W613*	Very severe		
30 g^#^	c.1388_1406delGGAGGGGGGTTTTCATCAC p.G463Rfs*7	Very severe	c.2238G > C p.W746C	Potentially mild		
31 g^#^	c.1388_1406del GGAGGGGGGTTTTCATCAC p.G463Rfs*7	Very severe	c.2238G > C p.W746C	Potentially mild		
32^#^	c.1561G > A p.E521K	Potentially less severe	c.2161G > T p.E72*	Very severe		
33^#^	c.2238G > C p.W746C	Potentially mild	c.2297A > C p.S7668	Potentially less severe		
34^#^	c.1657C > T p.Q553*	Very severe	c.2238G > C p.W764C	Potentially mild		
35^#^	c.827_845delTCACCCTGTGGAACCGGGA p.I276T*fs* *31	Very severe	c.2238G > C p.W764C	Potentially mild		
36^#^	c.2130C > G p.Y710*	Very severe	c.2167G > A V723M	Unknown		
37^#^	c.1309C > T p.R437C	Less severe	c.1822C > T p.R608*	Very severe		
38^#^	c.1114C > G p.H372D	Potentially less severe	c.1316 T > A p.M439K	Potentially mild		
39^#^	c.1082C > G p.P361R	Potentially less severe	c.1082C > G p.P361R	Potentially less severe		
40^#^	c.2662G > T p.E888*	Very severe	c.1409A > G p.N470S	Unknown		
41^#^	c.1465G > A p.D489N	Potentially less severe	c.2238G > C p.W746C	Potentially mild		
42	c.1082C > G p.P361R	Potentially less severe	c.2238G > C p.W746C	Potentially mild		
43	c.796C > T p.P266S	Potentially mild	c.1634C > T p.P545L	Less severe		
45	c.2238G > C p.W746C	Potentially mild	c.784G > A p.E262K	Potentially less severe		
46	c.2238G > C p.W746C	Potentially mild	c.784G > A p.E262K	Potentially less severe		
47	c.1562A > T p.E521V	Unknown	c.2238G > C p.W746C	Potentially mild		
48	c.503G > C p.R168P	Unknown	c.2238G > C p.W746C	Potentially mild		
52	c.1052_1076del23insG p.V351Gfs*33	Very severe	c.1622C > T p.P541L	Very severe		
53	NA	NA	NA	NA		
54	c.1622C > T p.P541L	Very severe	c.2399G > T p.S800I	Potentially less severe		
57	c.1798C > T p.R600C	Less severe	c.1557G > A p.M519I	Potentially less severe		
58	c.2173C > T p.R725W	Less severe	ND	NA		
59	c.1634C > T p.P545L	Less severe	c.1004G > A pG335E	Potentially less severe		
61	c.2040G > T p.L680L	Less severe	c.2238G > C p.W746C	Potentially mild		
62	c.2238G > C p.W746C	Potentially mild	c.1222A > G p.M408V	Potentially less severe		
67^#^	c.1309C > T p.R437C	Less severe	c.1309C > T p.R437C	Less severe		
68	c.241C > T pQ81*	Very severe	c.1309C > T p.R437C	Less severe		
69	c.2132_2133delCAinsGG p.T711R	Very severe	c.1669A > T p.I557F	Very severe	c.1409A > G p.N470S	Unknown
70	c.2237G > A p.W746*	Very severe	c.1871_1872delTCinsAG p.L624Q	Very severe		
71	c.-32-13 T > G p?	Potentially mild	c.503G > C p.R168P	Unknown		
72	c.1280 T > C p.M427T	Unknown	c.1222A > G p.M408V	Potentially less severe		
73	c.546G > T p.T182T	Potentially mild	c.2024-2026delACA p.675 p.675*del*N	Very severe		
74	c.2238G > C p.W746C	Potentially mild	c.2662G > T p.E888*	Very severe		
76	c.1622C > T p.P541L		c.1320_1322delATG p.Met440del	Potentially less severe		
77	c.2238G > C p.W746C	Potentially mild	c.2662G > T p.E888*	Very severe		
78	c.2238G > C p.W746C	Potentially mild	c.2238G > C p.W746C	Potentially mild		
80	c.2238G > C p.W746C	Potentially mild	c.2237G > T p.W746L	Potentially less severe	c.2210C > A p.T746N	Potentially less severe
81	c.2238G > C p.W746C	Potentially mild	c.2237G > C p.W746S	Potentially less severe		

NA, not available; ND, not detected; ^#^Passed away; a-g, The same letter means siblings; The non-pathogenic variant c.2065G > A p.E689K was detected in patient 44,67,68,78,80; the non-pathogenic variant c.752C > T P.S251L was detected in patient 75; the non-pathogenic variant c.1726G > A p.G576S was detected in patient 4,6,30,31,35,44,67,68,78,80; *Predicted severity of variants is according to the guidelines described in Kroos (15, 18, 19).

The most frequent mutation in the *GAA* gene was c.2238G > C (p.W746C), observed at an allele frequency of 21.6% (29/134) and in 43.3% of patients (29/67). The second most frequent variant was c.2662G > T (p.E888*fs*), with an allele frequency of 4.5% (6/134). According to the Human Gene Mutation Database (HGMD), five novel point variants were identified: c.2399G > T p.S800I, c.2130C > G p.Y710*fs*, c.1622C > T p.P541L, c.1327-7 T > A p.?, and c.1139C > T p.S380F. Several patients carried non-pathogenic variants, such as c.1726G > A p.G576S (in patients 4, 6, 30, 31, 35, 67, 68, 78, and 80) and c.2065G > A p.E689K (in patients 67, 68, 78, and 80).

### 3.5 Follow-up data

**Figure 4:**
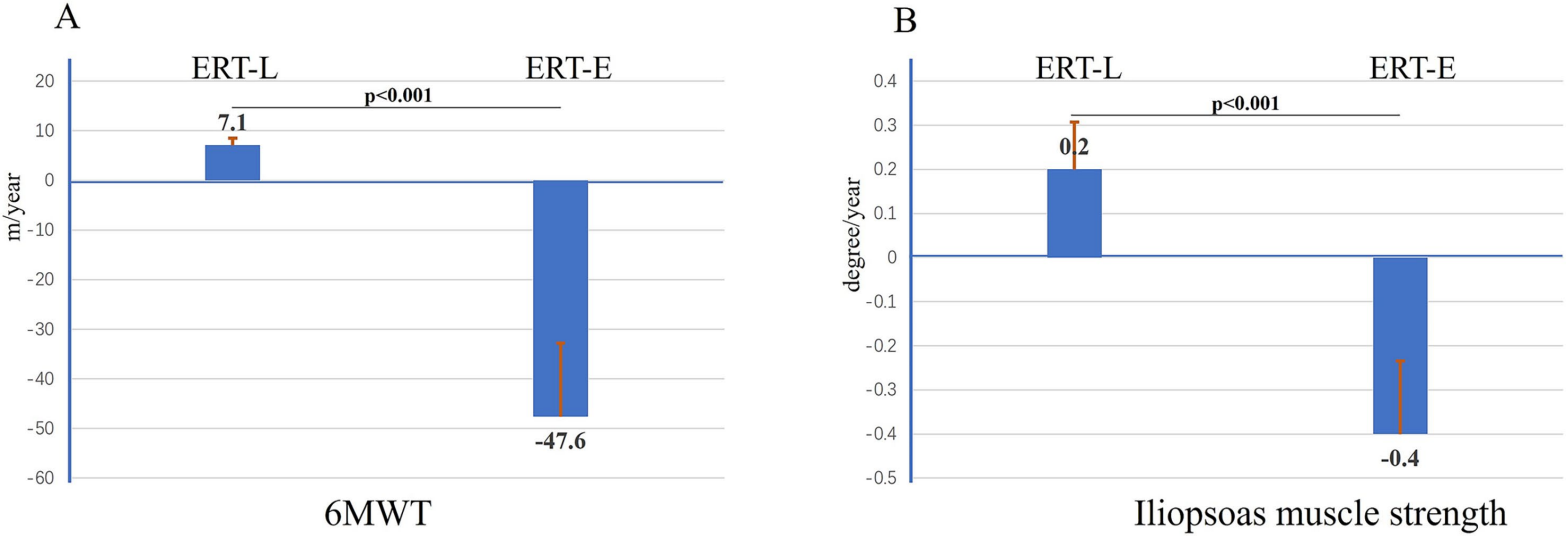
The difference between ERT-late and ERT-early group on 6MWT and iliopsoas muscle strength per year. **(A)** The 6MWT ability decreased as 7.1 meters/year in ERT-L group (*N* = 3); the 6MWT ability increased as 47.6 meters/year in ERT-E group (*N* = 7, *p* < 0.001); **(B)** The strength of iliopsoas muscle decreased as 0.2 degree/year in ERT-L group (*N* = 3); the strength of iliopsoas muscle increased as 0.4 degree/year in ERT-E group (*N* = 7, *p* < 0.001).

A cohort of 10 LOPD patients receiving ERT (with a dosage of 20 mg/kg every other week) was followed up for periods ranging from 1 to 10 years. Among these, ERT-E (early initiation: within one year of diagnosis, *n* = 7) and ERT-L (late initiation: after more than one year or treatment duration <50% of disease duration, *n* = 3) were defined. Specifically, 3 patients initiated ERT later in the disease duration, with ERT treatment duration constituting less than half of their total disease duration (designated as the ERT-L group), while 7 patients commenced ERT within one year of diagnosis (designated as the ERT-E group). In an exploratory subgroup analysis with very small sample sizes (ERT-E, *n* = 7; ERT-L, *n* = 3),comparisons between the two groups revealed that the 6MWT distance decreased by 7.1 meters per year in the ERT-L group, while it increased by 47.6 meters per year in the ERT-E group (*p* < 0.001, Figure 4A). Similarly, iliopsoas muscle strength declined by 0.2 degrees per year in the ERT-L group, but improved by 0.4 degrees per year in the ERT-E group (*p* < 0.001, Figure 4B). These findings are preliminary and should not be used to guide clinical decisions without confirmation in larger cohorts.

## 4 Discussion

This real-world, retrospective study elucidated the clinical, pathological, and genetic characteristics of 68 Chinese patients with LOPD from a single center over a longitudinal period of 6 years.

The clinical features indicated that the mean onset age was 16.0 years, with 9 patients exhibiting an onset age earlier than 3 years. The predominant clinical manifestation was symmetrical weakness in the proximal lower limbs; only 2 patients presented with asymmetrical weakness, and one patient did not exhibit muscle weakness. Respiratory failure (16 out of 68 cases) could be an initial symptom, potentially necessitating life support. The cohort exhibited a low BMI, averaging 17.9, indicative of underweight status. A lower BMI is associated with severe respiratory dysfunction in other studies (20, 21). However, BMI alone is an inadequate marker of nutritional status in neuromuscular diseases, as it does not distinguish fat from lean mass (22). In the absence of body composition data (e.g., DEXA, BIA), our findings regarding BMI should be interpreted with caution. Long-term follow-up is essential for continuous observation of the relationship between body composition and ERT outcomes.

Our study provides the first comprehensive analysis from a single-center cohort in China, highlighting a mortality rate of 22.1% among LOPD patients. This finding underscores the critical need for improved diagnostic protocols and timely intervention strategies, consistent with previous reports that early initiation of ERT significantly improves patient survival and quality of life (12, 23). Moreover, our results align with those from Taiwan, which reported a mortality rate of approximately 25%, indicating similar healthcare challenges in the region that warrant further investigation (24).

Among the 47 patients who received enzyme replacement therapy (ERT), the mortality rate was 6.4% (3/47), significantly lower than the 57.1% (12/21) observed in those without ERT. While ERT was associated with higher survival probability in this cohort, significant survival and selection biases limit any causal interpretation. Prior to 2020, patients could not immediately commence ERT upon diagnosis due to its introduction into China in May 2017 and subsequent inclusion in medical insurance coverage in 2020. Consequently, patients who deteriorated or died before 2020 were by definition assigned to the non-ERT group, introducing a severesurvival bias. Therefore, the observed difference in mortality between ERT and non-ERT groups should be viewed as descriptive only, not as evidence of treatment efficacy.

Prognostic evaluations from studies, particularly in Taiwan, indicate a stark contrast in survival rates between treated and untreated patients, with ERT recipients exhibiting a survival rate of 75% compared to just 40% for those not receiving treatment (11). This underscores the necessity for ongoing research to identify factors influencing survival rates, including access to ERT in low-resource settings and potential gaps in healthcare delivery systems that could affect patient prognosis (16). Future studies should aim to refine prognostic models to better predict patient outcomes based on a range of clinical and genetic factors.

The presence of advanced histopathological changes is strongly correlated with elevated mortality rates, indicating that the severity of muscle pathology should be integrated into patient management strategies (14). The identification of biomarkers predictive of muscle pathology can enable timely intervention and individualized treatment planning, thereby enhancing patient prognosis and quality of life (12). Our previous research has demonstrated that glycogenin reflects residual glycogen generated through cytosolic glycogenolysis, which co-localizes with Lamp1, suggesting that this residual glycogen is transported into lysosomes (data not published). We observed glycogenin-positive muscle fibers at the early stage of disease progression in a small number of patients (*n* = 3), while autophagy markers remained negative. These observations generate the hypothesis that glycogenin expression may serve as an early pathological marker, but this hypothesis requires validation in larger, prospective longitudinal studies. Furthermore, it is consistent with our prior hypothesis that residual glycogen from incomplete glycogenolysis may enter lysosomes and accumulate (25). Given the limited sample, these findings should be considered hypothesis-generating, not conclusive.

Although morphological and autophagic improvements were observed in pre- and post-ERT biopsies, pathological changes in the natural course of the disease without ERT administration have rarely been reported (26). The three patients who underwent re-biopsy showed varying degrees of pathological deterioration. The muscle pathology features of LOPD include fiber size variation, significant presence of small basophilic particles within muscle fibers, RVs and cytoplasmic bodies observed on MGT, glycogen accumulation as shown by PAS staining, type II fiber atrophy, vacuolation in type II fibers, and strong positive ACP staining. In three patients who underwent a second muscle biopsy prior to ERT, the extent of muscle pathology deterioration varied considerably. Despite similar mutation types (point mutations with comparable predicted protein function), the differences in pathology may be attributed to lifestyle factors such as diet, stress, or exercise. The correlation analysis revealed that the severity of muscle pathology in LOPD is associated with lower BMI, shorter 6MWT and spinal curvature abnormalities, as these factors are interrelated with muscle strength. Notably, the severity of muscle pathology did not correlate with patient age, disease duration at biopsy, residual GAA enzyme activity, CK levels results, or iliopsoas muscle strength. These negative correlation findings are consistent with previous reports (27).

Genetic analysis revealed that compound heterozygotes are the most common form of variation (63/67) and most of the variants have already been recorded in the database (15, 18, 19). The most frequent mutation of *GAA* gene in this cohort was c.2238G > C (p.W746C), observed in 43.3% of patients (29/67). The c.2238G > C mutation has been reported as a common mutation in Chinese LOPD with an allele frequency 27.08% (13/48) (28) and a patient frequency 58.1% (18/31) (29). This was consistent with our findings, where the allele frequency was 21.6% (29/134). Additionally, this mutation has been sporadically reported in other countries, including Malaysia (30), Korea (31), Belgium (32), Japan (33) and the United States (34).

During the follow-up study, 10 LOPD patients receiving ERT treatment were regularly monitored at our center. In an exploratory analysis (ERT-E n = 7, ERT-L *n* = 3), we observed that patients who initiated ERT shortly after diagnosis exhibited greater improvements in muscle strength and 6MWT results compared to those who started treatment later. However, the very small sample size (especially in the ERT-L group) precludes any definitive conclusion. These preliminary findings should be interpreted with caution and require replication in larger cohorts.

ERT has been demonstrated to enhance 6MWT performance and respiratory function in LOPD patients based on a substantial body of clinical evidence (moderate-certainty evidence) (35). Previous studies have demonstrated that patients undergoing ERT may exhibit varying degrees of improvement (36). Treated patients generally showed enhanced muscle strength (8). However, post-treatment muscle pathology worsened in some patients due to delayed diagnosis (26). Our data do not allow robust comparison between early and late initiation.

This study has several important limitations. First, the survival comparison between ERT and non-ERT groups is subject to severe survival and selection bias due to the timing of ERT availability in China. Second, all reported correlations are based on univariate analyses without adjustment for confounding variables; multivariate regression was not feasible due to limited sample size and missing data. Third, the subgroup analysis of ERT timing included only 10 patients (3 in the late group), making it underpowered. Fourth, we lacked body composition data (DEXA, BIA) and detailed respiratory assessments (supine FVC, diaphragmatic weakness, NIV use). Fifth, the conclusion regarding glycogenin as an early biomarker is based on a very small number of longitudinal samples and should be considered hypothesis-generating. Therefore, all findings should be interpreted as exploratory and descriptive.

In summary, this real-world retrospective study describes clinical, pathological, and genetic features of 68 Chinese LOPD patients. ERT was associated with higher survival probability, but survival bias precludes causal inference. Early ERT initiation showed an association with better functional outcomes in a small exploratory subgroup. Lower BMI, shorter 6MWT, and spinal curvature abnormalities correlated with more severe muscle pathology in univariate analyses. The c.2238G > C (p.W746C) mutation was most common. All findings are hypothesis-generating due to the study’s observational design, biases, and small sample sizes. Future prospective studies with multivariate analyses and longer follow-up are needed.

## Data Availability

The original contributions presented in the study are included in the article/Supplementary material, further inquiries can be directed to the corresponding authors.

## Supplementary material

SUPPLEMENTARY FIGURE S1 The criteria for severity of muscle pathology in LOPD.
