# Supplementary figures and images for: Clinical, pathological and genetic features as well as follow-up of 68 patients with late-onset Pompe disease: a single-center retrospective study
Source: Front Nutr. 2026 Apr 28;13:1797345. doi: 10.3389/fnut.2026.1797345 (PMC13160774; doi:10.3389/fnut.2026.1797345)

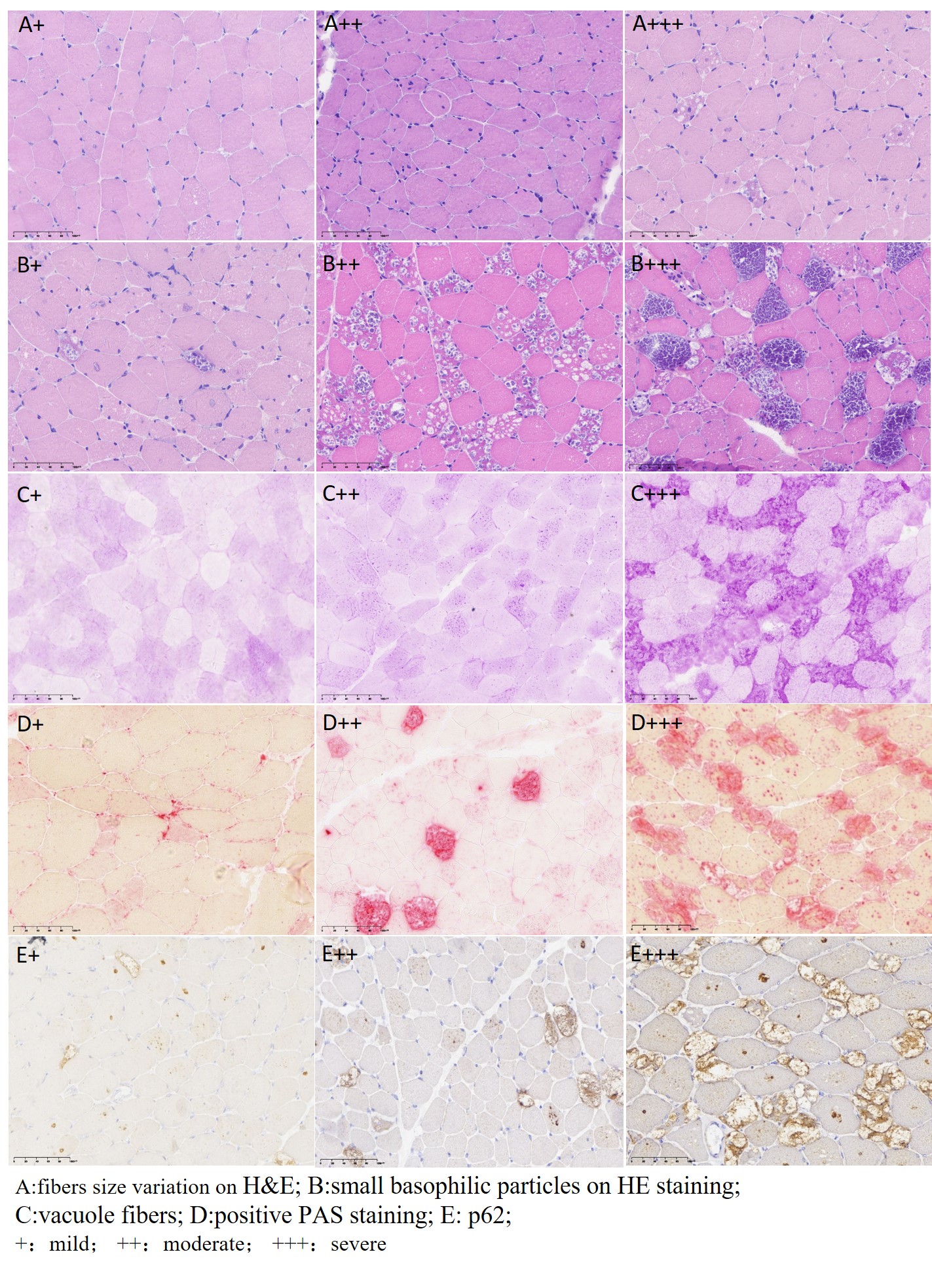

Supplement: SUPPLEMENTARY FIGURE S1 — The criteria for severity of muscle pathology in LOPD. [file Image_1.jpg]
